# Chronic Pelvic Inflammation Diminished Ovarian Reserve as Indicated by Serum Anti Mülerrian Hormone

**DOI:** 10.1371/journal.pone.0156130

**Published:** 2016-06-06

**Authors:** Linlin Cui, Yan Sheng, Mei Sun, Jingmei Hu, Yingying Qin, Zi-Jiang Chen

**Affiliations:** 1 Center for Reproductive Medicine, Provincial Hospital Affiliated to Shandong University, Jinan, 250001, China; 2 The Key laboratory for Reproductive Endocrinology of Ministry of Education, Jinan, China; 3 Shandong Provincial Key Laboratory of Reproductive Medicine, Jinan, 250001, China; 4 National Research Center for Assisted Reproductive Technology and Reproductive Genetics, Jinan, 250001, China; 5 Center for Reproductive Medicine, Ren Ji Hospital, School of Medicine, Shanghai Jiao Tong University, Shanghai, 200000, China; Shanghai Key Laboratory for Assisted Reproduction and Reproductive Genetics, Shanghai, 200000, China; China Agricultural University, CHINA

## Abstract

**Objective:**

To explore the potential damaging effect of chronic pelvic inflammation on ovarian reserve.

**Design:**

Case-control study.

**Patients:**

A total of 122 women with bilateral tubal occlusion, diagnosed by hysterosalipingography (HSG) and 217 women with normal fallopians were recruited.

**Measurements:**

Serum anti-Mullerian hormone (AMH), basic follicle-stimulating hormone (FSH), luteining hormone (LH), estradiol (E_2_), and testosterone (T) were measured; and antral follicle counts (AFCs) were recorded.

**Results:**

Significantly lower level of AMH was observed in women with bilateral tubal occlusion compared to control group [2.62 (2.95) ng/ml vs. 3.37 (3.11) ng/ml, P = 0.03], and the difference remained after adjustment of BMI (P_adjust_ = 0.04). However, no statistical difference was found in the levels of FSH [7.00 (2.16) IU/L vs. 6.74 (2.30) IU/L], LH [4.18 (1.52) IU/L vs. 4.63 (2.52) IU/L], E_2_ [35.95 (20.40) pg/ml vs. 34.90 (17.85) pg/ml], T [25.07±11.46 ng/dl vs. 24.84±12.75 ng/dl], and AFC [6.00 (4.00) vs. 7.00 (4.00)] between two groups (p>0.05).

**Conclusions:**

Women with bilateral tubal occlusion showed decreased AMH level, suggesting that chronic pelvic inflammation may diminish ovarian reserve. More caution should be paid when evaluating the detriment of PID on female fertility.

## Introduction

Pelvic inflammatory disease (PID), responded to infection, irritation, or injury, has become a public health problem because of its deleterious effects on female reproductive system. Fallopian tubal occlusion, responsible for 42.3% of female infertility, is the commonest sequela of PID. It has been reported that 61.1% of infertile women with tubal factor resulted from non-specific PID [1].

As is well known, PID has direct deleterious effect on pelvic environment and endometrial receptivity However, it is unclear whether chronic pelvic inflammation will destroy ovarian reserve and impair follicles quantitatively and qualitatively. Patients with Crohn’s disease or dermatomyositis had decreased anti-Müllerian hormone (AMH) and antral follicle count (AFC) [2, 3], which suggested that inflammation adversely affect ovarian reserve. However, there is no clear mechanism elucidating the role of inflammation on follicle.

Here, we compared the basal FSH, estradiol (E_2_), testosterone (T), AMH and AFC between patients with tubal occlusion and normal fallopian to explore the potential effect of chronic pelvic inflammation on ovarian reserve.

## Materials and Methods

### Participants

A total of 122 women with bilateral tube occlusion diagnosed by hysterosalipingography (HSG) were enrolled. Other causes of infertility, such as ovulatory dysfunction, endocrinopathy, male factor, have been excluded. 217 age-matched women, asking for infertility treatment because of male factor, with normal fallopian tubes were recruited as controls. All subjects were from the Center for Reproductive Medicine, Shandong University. Women with abdominal or pelvic surgery, other disorders affecting ovarian reserve, including endometriosis, thyroid dysfunction, hyperprolactinemia, polycystic ovary syndrome (PCOS), premature ovarian insufficient (POI), and exposure to chemotherapy or radiotherapy previously were excluded.

### Measurements

All subjects were subjected to anthropometric data, including height and weight. Body mass index (BMI) was calculated using weight (kg)/height^2^ (m^2^). Menstrual cycle, surgical history, and other diseases were recorded. Trans-virginal ultrasound was performed to evaluate AFC and hysterosalpingogram (HSG) to evaluate the status of fallopian tubes.

Blood was sampled after 8 hours of fasting on day 3 to day 5 of menstruation for measuring AMH, FSH, luteinizing hormone (LH), E_2_, and T, using enzyme-linked immunosorbent assay (Ansh Labs Webster, USA; for AMH) and chemiluminescence immunoassays (Roche Diagnostics, Germany; for other serum parameters), respectively, all with intra- and inter-assay coefficients of variation<10%. All the data were listed in S1 Table.

### Ethical approval

Written informed consents were obtained from all participants, and the study was approved by the Institutional Review Board of Reproductive Medicine of Shandong University.

### Statistics

Data analysis was performed using Statistical Package for the Social Sciences for Windows (version 20.0; SPSS Inc., Chicago, IL, USA). Normality test was performed first for continuous variables of both groups using Kolmogorov-Smirnov test. Data were presented as mean± standard deviation for normality distribution variables and median(quartile interval) for non-normality distribution variables. Lg-transformation was used in comparison of the latter. Difference of the means between two groups was determined using Students’ T test. Analysis of covariance (ANCOVA) was used for adjustment of the covariance. Chi-square analysis was used for categorical variables comparison. Statistical significance was set at P<0.05.

## Results

Clinical characteristics of all participants were presented in Table 1. The ages [31.43±4.94 yrs vs. 30.85±5.11 yrs, P = 0.31] and prevalence of childbearing history [51.60% vs. 45.60%, P = 0.29] between two groups were comparable. Compared with controls, patients with tube obstruction had higher BMI [22.89(4.44) kg/m^2^ vs. 21.64(4.12) kg/m^2^, P = 0.03].

**Table 1:** Clinical characteristics.

	Bilateral tube occlusion (n = 122)	Normal fallopian tube (n = 217)	P
Age (yrs)	31.43±4.94	30.85±5.11	0.31
**BMI**^a^ **(kg/m**^**2**^**)**	**22.89 (4.44)**	**21.64 (4.12)**	**0.03**
Childbearing [%(N)]	51.60 (63)	45.60 (99)	0.29

Data were presented as mean± standard deviation for age, median (interquartile range) for BMI, and incidence (positive numbers) for categorical variable.

BMI: body mass index.

^a^: Statistical significance was found between cases and control subjects(P<0.05).

Lower AMH level was observed in patients with bilateral tube occlusion than controls [2.62(2.95)ng/ml vs. 3.37(3.11)ng/ml, P = 0.03], even after adjustment with BMI [P_adjust_ = 0.04]. However, no difference was found in AFC, FSH, LH, E_2_, and T between two groups (Table 2).

**Table 2:** Comparison of parameters indicating ovarian reserve between cases and controls.

	Bilateral tube occlusion (n = 122)	Normal fallopian tube (n = 217)	P	P_adjust_
AFC	6 (4)	7 (4)	0.23	0.18
FSH(IU/L)	7.00 (2.16)	6.74 (2.30)	0.77	0.99
LH (IU/L)	4.18 (1.52)	4.63 (2.52)	0.30	0.58
E_2_(pg/ml)	35.95 (20.40)	34.90 (17.85)	0.42	0.20
T(ng/dL)	25.07±11.46	24.84±12.75	0.87	0.99
**AMH**^a^**(ng/ml)**	**2.62 (2.95)**	**3.37 (3.11)**	**0.03**	**0.04**

Data were presented as mean± standard deviation for normality distribution variables and median(quartile interval) for non-normality distribution variables.

Difference of the means between two groups was determined after lg-transformation using Students’ T test. Analysis of covariance (ANCOVA) was used for adjustment of the covariance.

FSH: follicle stimulating hormone; LH: luteinizing hormone; E_2_: estradiol; T: testosterone; AMH: anti-müllerian hormone

P_adjust_: adjustment for BMI

^a^: Statistical significance was found between cases and control subjects(P<0.05).

## Discussion

AMH was exclusively produced by granulosa cells of preantral and small antral follicles[4], and was an sensitive indicator for ovarian reserve. In the present study, lower AMH level was observed in women with bilateral tube occlusion compared with normal tubes. It indicated a potential adverse effect of chronic pelvic inflammation on ovarian reserve.

Changes in volumetric density of the arteries and veins of the microcirculatory bed in the ovary and uterine tube have been observed in rats with aseptic inflammation after application of spherical carbonate-mineral sorbent[5]. The index of blood flow was turn out to be correlated with the incidence of apoptotic granulosa cells[6]. Thus, the decrease of effective granulosa cells may reduce the secretion of AMH in patients with pelvic inflammation through damaged blood flow. Chronic inflammation accompanied with not only changing in blood supply but also possible excessive DNA damage[7, 8]. Oocytes are particularly vulnerable to ubiquitous external damage. Subsequently, the number of damaged follicles may increase, leading to a greater rate of follicle loss through atresia and a decrease of AMH[9, 10]. Besides, the dysfunction in steroidogenesis synthesis of granulosa cells due to inflammatory response may also be an extra underlying mechanism[11–14].

AFC is another common indicator for ovarian reserve besides AMH. The correlation between AMH and AFC has been indicated in previous study[15]. In the present study relative fewer AFC was found in case group than controls but not reaching statistical difference. Distinct sensitivity may be one of the explanations. Compared with AFC, the level of AMH not only represents the number of small antral follicles but also preantral follicles, which are invisible by ultrasound scan. Therefore, compared with AFC, AMH is much earlier and more sensitive for assessing follicular pool.

The present study firstly explored the potential deleterious effect of chronic pelvic inflammation on ovarian reserve indicated by AMH. Several limitations exist. Firstly, fallopian tube inflammation may not be totally diagnosed by HSG. As the severest sequel of pelvic inflammation, bilateral tube occlusion could represent the serious effect of PID on ovarian reserve. Changes of ovarian reserve in other patient group should be further validated, for example PID diagnosed by laparoscope. Secondly, difference in BMI between two groups existed. BMI is inverse related with ovarian reserve, and usually but not always, in patients with PCOS[16–19]. Therefore, we performed further analysis using ANCOVA to adjust BMI. And the difference in AMH level between two groups remains. Thirdly, relatively small sample size limited the statistical power, to some extent.

## Conclusion

Bilateral tubal occlusion showed decreased AMH level, suggesting that chronic pelvic inflammation may diminish ovarian reserve. Therefore, more caution should be paid when evaluating the detriment of PID on female fertility.

## Supporting Information

S1 Table Original data. (PDF)
