# Supplementary material for: Chronic Pelvic Inflammation Diminished Ovarian Reserve as Indicated by Serum Anti Mülerrian Hormone
Source: PLoS One. 2016 Jun 6;11(6):e0156130. doi: 10.1371/journal.pone.0156130 (PMC4894572; doi:10.1371/journal.pone.0156130)
Supplement: S1 Table — (PDF) [file pone.0156130.s001.pdf]

S1 Table Original data

ID	Group(0=control,1=case)	Age	BMI	Previous pregnancy	AFC	FSH	LH	E2	T	AMH
1	0	25	17.72	0	8	9.72	17.00	73.13	0.22	7.14
2	0	40	23.44	1	10	6.11	5.88	77.30	12.86	3.68
3	0	30	24.80	0	5	5.29	7.89	72.00	21.32	1.40
4	0	39	24.80	1	4	7.48	3.81	71.60	21.46	0.24
5	0	28	21.64	0	5	5.25	5.33	70.30	14.75	3.88
6	0	27	19.95	1	11	5.41	4.22	69.70	26.23	8.12
7	0	32	22.48	0	3	6.97	4.94	65.40	22.51	1.47
8	0	33	16.73	1	8	8.00	9.00	65.00	52.00	2.39
9	0	23	25.22	0	7	6.29	5.55	63.80	40.14	3.04
10	0	31	21.88	0	8	5.22	5.63	63.20	23.55	4.14
11	0	24	20.72	0	9	7.51	4.99	62.90	23.92	2.09
12	0	32	18.87	1	3	5.86	4.05	62.00	34.34	0.60
13	0	42	26.64	1	4	5.34	1.57	60.90	32.48	0.92
14	0	32	20.96	0	12	5.00	3.83	60.40	41.40	6.90
15	0	38	20.76	1	5	8.06	5.95	60.20	20.82	3.39
16	0	47	24.44	1	4	9.90	2.49	57.50	11.33	2.65
17	0	26	20.20	0	11	6.12	5.18	57.00	16.80	3.37
18	0	40	21.99	1	4	10.15	9.39	56.80	7.36	1.80
19	0	33	22.96	1	6	4.00	2.03	56.00	14.99	2.57
20	0	36	19.92	1	4	8.89	3.63	56.00	21.56	0.50
21	0	38	28.44	1	7	8.00	4.00	56.00	25.00	3.53
22	0	27	17.45	1	6	7.15	5.10	55.70	27.61	3.59
23	0	28	18.22	0	5	4.22	3.46	55.30	16.76	0.95
24	0	31	23.44	0	9	5.69	4.38	54.70	33.95	9.02
25	0	26	21.05	1	9	5.00	5.00	53.00	31.00	5.41
26	0	36	21.48	1	6	7.43	4.46	52.90	29.12	3.27
27	0	35	21.50	0	9	7.02	1.87	52.50	26.05	9.04
28	0	35	20.31	1	4	5.65	6.40	52.30	21.87	1.36
29	0	29	20.06	1	10	7.00	6.00	52.00	25.00	1.42
30	0	26	19.14	0	8	4.95	3.44	51.70	27.59	4.03
31	0	40	23.80	1	3	10.30	5.90	51.20	16.48	1.67
32	0	29	21.63	0	3	8.78	3.82	50.50	20.07	1.96
33	0	30	26.37	0	11	3.53	2.69	50.40	38.50	14.94
34	0	32	21.48	1	10	4.00	3.00	50.00	30.00	3.76
35	0	27	21.45	0	7	6.69	5.37	49.70	39.48	2.68
36	0	35	22.77	0	4	7.66	7.22	49.30	3.41	2.01
37	0	30	18.36	0	7	7.32	11.27	48.70	19.30	3.38
38	0	27	21.88	0	11	4.83	3.50	48.50	14.26	4.72
39	0	24	25.24	0	9	5.58	2.57	48.40	53.22	5.64
40	0	30	20.57	0	9	6.66	5.24	48.30	25.71	4.00
41	0	31	21.45	1	5	8.00	4.00	48.00	17.00	0.62
42	0	34	26.44	0	7	6.25	3.15	47.10	20.59	5.15
43	0	36	21.22	0	5	12.00	4.00	47.00	26.00	3.53
44	0	27	20.20	0	8	7.37	3.74	46.80	9.30	0.50
45	0	33	18.43	0	5	6.51	3.07	46.10	29.06	0.90
46	0	29	23.98	1	3	9.01	3.49	45.80	11.99	4.46
47	0	23	22.19	0	6	9.57	4.22	45.30	19.96	1.15
48	0	33	19.60	1	9	5.68	5.94	45.30	39.26	5.61
49	0	33	19.49	1	4	9.68	6.02	45.20	17.19	2.19
50	0	36	20.64	1	8	6.81	6.71	45.00	12.22	0.21

51	0	44	23.44	1	6	6.19	4.84	44.90	22.56	1.38
52	0	31	20.83	0	7	7.18	6.76	44.70	13.59	3.62
53	0	35	18.67	0	3	5.96	2.08	44.20	19.56	0.71
54	0	33	23.88	0	4	8.69	4.78	44.10	25.11	0.71
55	0	27	24.84	0	8	5.26	3.74	43.60	76.70	2.53
56	0	23	17.97	0	9	5.84	5.04	43.50	16.07	3.78
57	0	30	22.03	1	10	4.00	3.00	43.00	53.00	3.53
58	0	32	20.62	0	9	7.38	7.40	43.00	39.97	6.54
59	0	25	18.37	0	11	8.92	6.12	42.50	34.66	8.29
60	0	34	20.83	0	5	13.19	6.15	42.20	21.69	1.63
61	0	28	20.81	0	9	10.47	6.69	42.10	37.32	10.00
62	0	24	19.53	1	10	8.00	7.00	42.00	45.00	3.10
63	0	25	24.22	1	7	6.00	3.00	42.00	52.00	8.56
64	0	28	20.31	1	12	7.00	5.00	42.00	59.00	3.53
65	0	37	21.09	0	5	10.69	3.80	41.80	29.37	2.22
66	0	37	22.27	0	6	5.78	5.86	41.80	29.80	3.79
67	0	29	20.19	0	5	8.75	2.92	41.60	2.50	3.22
68	0	26	19.53	0	7	6.63	9.55	41.20	25.79	5.51
69	0	39	21.29	1	5	5.99	10.13	41.10	20.94	3.19
70	0	39	25.22	1	3	5.25	3.51	41.00	33.90	0.46
71	0	32	19.92	0	8	5.91	3.46	40.90	24.61	2.00
72	0	33	20.03	0	9	4.14	1.57	40.90	24.75	3.19
73	0	27	24.61	0	4	6.14	2.86	40.70	43.64	0.78
74	0	28	21.09	0	11	5.13	6.15	40.40	26.96	8.16
75	0	29	25.04	0	8	5.52	7.76	40.40	23.92	0.30
76	0	24	20.96	0	9	5.80	4.78	40.10	22.72	5.75
77	0	26	24.03	0	6	5.75	3.62	40.00	34.94	10.63
78	0	26	21.28	0	11	6.17	7.63	40.00	26.63	2.65
79	0	43	20.96	1	5	7.79	3.20	39.50	16.65	1.46
80	0	23	19.48	0	6	6.33	8.00	39.40	23.47	8.10
81	0	36	23.34	0	4	7.44	2.41	39.20	49.72	2.41
82	0	27	18.82	0	6	6.67	3.48	39.10	21.54	2.82
83	0	32	23.59	1	8	5.00	3.00	39.00	52.00	1.86
84	0	25	28.26	1	10	4.59	3.55	38.80	68.64	3.94
85	0	32	25.08	0	10	7.78	7.84	38.60	39.39	11.20
86	0	37	19.88	1	3	10.10	4.47	38.50	22.18	0.72
87	0	34	24.14	0	7	6.43	4.89	38.40	9.44	2.66
88	0	29	23.74	1	4	9.16	4.12	38.30	14.43	2.76
89	0	28	22.96	0	10	6.10	3.68	38.10	16.78	5.64
90	0	26	19.43	1	6	7.00	6.00	38.00	22.00	6.05
91	0	32	18.42	1	6	7.00	4.00	38.00	28.00	3.53
92	0	33	24.14	0	9	5.71	3.23	38.00	18.71	5.89
93	0	25	21.64	0	8	5.41	2.82	37.90	32.41	2.25
94	0	40	21.48	1	4	11.34	4.82	37.40	7.32	0.28
95	0	31	23.44	1	7	8.00	3.00	37.00	43.00	3.53
96	0	42	20.26	1	2	14.22	6.43	36.80	13.21	0.51
97	0	33	18.29	0	6	8.71	4.94	36.70	18.45	0.59
98	0	30	18.75	0	8	7.09	4.44	36.20	20.10	5.06
99	0	33	23.31	1	6	11.17	5.85	36.20	7.55	1.81
100	0	36	23.31	0	4	6.13	3.19	36.20	53.19	4.19
101	0	37	19.81	1	7	4.80	3.59	36.10	10.40	4.30
102	0	24	19.53	0	6	6.00	5.00	36.00	17.00	2.53
103	0	35	20.00	1	6	9.00	4.00	36.00	27.00	2.00
104	0	23	21.19	0	8	7.30	6.93	35.50	11.42	2.79

105	0	44	23.03	1	6	5.92	4.50	35.40	19.79	2.02
106	0	27	22.66	0	4	7.25	3.96	35.30	11.01	2.40
107	0	23	20.31	0	7	8.16	6.50	35.20	28.89	1.64
108	0	39	24.54	1	7	6.75	6.73	35.00	39.00	2.06
109	0	30	21.88	0	6	5.52	4.09	34.90	17.06	3.64
110	0	29	18.67	1	9	8.67	5.82	34.50	15.54	1.45
111	0	33	19.65	1	5	11.02	5.40	34.40	30.30	1.39
112	0	40	22.41	1	4	12.31	6.55	34.20	26.32	0.50
113	0	29	18.37	0	6	7.69	6.91	34.00	39.11	5.16
114	0	35	20.31	0	8	9.00	4.00	34.00	30.00	2.03
115	0	39	24.03	0	8	8.00	7.00	34.00	33.00	9.92
116	0	29	25.00	0	10	4.63	6.44	33.90	27.71	4.68
117	0	23	25.59	0	8	6.00	5.23	33.80	16.47	4.98
118	0	32	27.55	0	8	11.39	8.28	33.80	33.26	2.16
119	0	25	19.05	0	6	6.04	6.86	33.40	30.29	3.64
120	0	26	20.20	0	6	6.61	2.91	33.20	8.34	0.77
121	0	34	21.26	1	2	6.00	4.00	33.00	18.00	0.68
122	0	31	25.53	0	8	6.05	1.92	32.70	23.88	4.12
123	0	32	20.28	0	7	5.40	3.97	32.70	23.55	2.19
124	0	36	28.28	1	4	7.44	4.84	32.70	7.73	4.45
125	0	30	19.81	0	13	4.37	2.71	32.40	25.29	11.07
126	0	32	23.73	1	7	7.26	4.85	32.30	11.37	1.48
127	0	26	23.71	1	13	5.00	8.00	32.00	46.00	0.66
128	0	29	24.46	1	6	6.39	4.08	32.00	18.41	2.75
129	0	30	21.23	1	10	7.00	3.00	32.00	31.00	3.99
130	0	33	21.48	0	5	10.00	5.00	32.00	20.00	1.06
131	0	25	24.80	1	4	6.31	3.07	31.70	14.55	1.46
132	0	26	21.26	0	16	4.79	5.45	31.20	32.50	4.14
133	0	31	22.43	1	6	5.70	4.15	31.20	17.01	2.15
134	0	30	21.61	1	8	8.00	4.00	31.00	33.00	1.54
135	0	36	21.48	0	7	5.55	6.40	31.00	22.47	0.92
136	0	32	22.10	0	3	21.32	9.21	30.50	21.74	2.28
137	0	24	20.93	1	6	8.00	6.00	30.00	52.00	2.13
138	0	29	30.00	0	11	5.00	4.00	30.00	32.00	8.00
139	0	30	20.43	0	10	6.00	4.00	30.00	26.00	3.53
140	0	31	20.81	1	13	5.00	5.00	30.00	14.00	5.46
141	0	36	27.55	1	6	8.06	3.25	30.00	31.01	4.80
142	0	39	25.34	1	7	11.45	5.84	29.80	17.29	0.73
143	0	31	19.93	1	11	5.00	4.00	29.00	29.00	6.94
144	0	31	22.84	1	10	7.00	3.00	29.00	24.00	3.53
145	0	33	23.88	1	8	6.00	6.00	29.00	19.00	5.43
146	0	28	21.45	0	9	7.87	6.36	28.60	31.62	7.83
147	0	24	18.73	1	12	6.00	8.00	28.00	29.00	13.52
148	0	25	20.31	1	8	8.00	8.00	28.00	24.00	1.16
149	0	27	23.03	0	6	6.00	4.00	28.00	43.00	3.13
150	0	28	21.51	1	8	6.00	3.00	28.00	34.00	2.83
151	0	31	24.84	1	12	4.00	6.00	28.00	35.00	3.53
152	0	32	21.00	1	5	8.00	6.00	28.00	14.00	5.00
153	0	32	26.67	0	7	8.00	4.00	28.00	14.00	4.03
154	0	32	21.48	1	5	6.00	6.00	28.00	43.00	3.53
155	0	29	32.46	0	7	7.45	5.24	27.80	9.63	0.55
156	0	26	23.03	0	10	7.08	5.22	27.70	25.94	4.80
157	0	34	20.31	1	6	10.64	5.88	27.10	14.90	3.80
158	0	26	20.51	1	5	5.00	4.00	27.00	12.00	1.47

159	0	30	27.34	1	8	4.00	2.00	27.00	32.00	0.59
160	0	35	24.80	1	5	7.65	2.35	26.70	13.61	0.70
161	0	33	24.89	0	8	6.28	2.53	26.20	14.20	8.39
162	0	26	20.55	0	6	9.18	9.72	26.10	40.27	5.83
163	0	32	21.23	0	7	8.01	5.28	26.00	6.59	5.48
164	0	32	24.41	1	9	5.00	5.00	26.00	29.00	0.91
165	0	30	21.26	0	9	4.90	3.09	25.70	39.37	5.41
166	0	31	23.61	0	4	5.08	2.01	25.60	31.39	1.43
167	0	41	30.86	0	8	6.74	4.15	25.60	3.93	2.12
168	0	24	26.57	0	18	6.80	4.68	25.50	35.19	8.10
169	0	37	26.22	0	5	4.72	4.01	25.30	49.82	2.05
170	0	25	18.67	0	8	7.40	4.67	25.00	27.21	10.91
171	0	27	24.03	1	9	7.00	3.00	25.00	16.00	3.85
172	0	28	23.23	1	10	5.00	4.00	25.00	11.00	7.46
173	0	40	26.03	1	10	5.07	3.79	25.00	8.11	0.78
174	0	39	24.44	0	6	4.76	1.19	24.90	16.94	2.38
175	0	27	20.20	0	10	8.33	5.02	24.70	18.90	0.27
176	0	30	21.51	0	9	11.11	6.32	24.40	30.87	6.62
177	0	24	17.91	0	10	7.87	6.38	24.30	22.86	4.71
178	0	24	25.22	1	13	7.00	5.00	24.00	26.00	3.53
179	0	26	20.03	0	8	10.00	4.00	24.00	24.00	3.53
180	0	26	19.92	1	7	6.00	7.00	24.00	31.00	3.53
181	0	34	22.06	1	6	11.00	3.00	24.00	18.00	0.65
182	0	24	23.05	0	12	7.11	2.45	23.90	24.85	4.82
183	0	29	22.39	1	10	9.00	8.00	23.00	17.00	3.53
184	0	35	19.49	1	6	6.00	9.00	23.00	4.00	3.37
185	0	23	21.72	0	5	9.73	3.30	22.00	23.99	3.56
186	0	30	20.00	0	6	8.00	2.00	22.00	10.00	1.00
187	0	33	28.98	1	6	8.00	4.00	22.00	23.00	1.78
188	0	25	24.98	0	10	5.37	2.66	21.50	43.56	7.37
189	0	37	25.10	0	6	8.99	3.32	21.40	21.10	4.16
190	0	23	23.63	0	11	6.33	6.10	21.30	33.61	8.34
191	0	26	29.24	0	10	5.29	2.86	21.30	31.29	8.49
192	0	25	30.49	1	11	6.00	5.00	21.00	14.00	3.13
193	0	29	21.48	1	10	8.00	7.00	21.00	10.00	3.53
194	0	31	20.70	1	11	7.00	7.00	21.00	30.00	3.53
195	0	32	25.51	0	9	7.00	4.00	21.00	28.00	2.24
196	0	25	25.39	0	15	4.87	2.61	20.60	18.13	8.21
197	0	44	25.51	1	4	9.49	6.76	20.50	21.72	0.22
198	0	32	21.88	1	9	6.51	3.47	19.90	16.88	12.00
199	0	40	25.91	0	5	7.74	4.59	18.80	4.03	2.80
200	0	26	22.31	1	10	5.00	7.00	18.00	19.00	4.55
201	0	30	19.05	1	10	7.00	6.00	18.00	31.00	5.04
202	0	33	24.03	1	6	9.48	5.78	17.40	16.75	1.89
203	0	28	20.58	1	8	7.28	8.87	17.20	19.40	8.77
204	0	27	19.53	1	7	6.00	3.00	17.00	13.00	3.53
205	0	28	21.88	0	9	6.20	9.88	15.85	0.21	4.39
206	0	25	22.23	0	13	6.55	6.64	15.60	14.58	5.56
207	0	26	26.00	0	13	4.00	3.00	15.00	43.00	3.00
208	0	38	19.10	0	9	9.88	5.60	13.90	15.84	5.61
209	0	31	26.23	1	12	6.21	3.20	13.70	23.78	1.00
210	0	27	25.71	1	8	6.00	8.00	12.00	39.00	3.53
211	0	30	24.77	0	4	5.25	4.12	11.40	22.61	2.87
212	0	34	20.43	1	5	6.46	4.63	11.10	2.50	2.19

213	0	29	24.44	0	8	5.41	4.52	10.80	16.78	6.32
214	0	22	19.53	0	8	9.30	8.06	10.10	10.78	3.88
215	0	34	23.83	1	8	7.23	3.05	9.80	16.71	2.29
216	0	32	36.72	0	7	7.16	4.12	9.30	66.09	0.44
217	0	31	26.17	0	8	7.00	4.00	7.00	14.00	1.73
218	1	33	29.14	0	4	4.00	18.00	76.00	17.00	1.25
219	1	34	21.88	0	10	7.00	5.00	71.00	28.00	2.27
220	1	40	20.31	0	4	4.00	4.00	72.00	24.00	1.28
221	1	30	21.34	0	12	9.18	8.04	72.70	2.50	1.54
222	1	39	22.58	1	4	8.00	4.00	72.00	13.00	0.38
223	1	32	20.08	1	7	7.46	3.89	70.70	29.39	0.89
224	1	27	19.10	1	8	4.00	5.00	68.00	34.00	7.34
225	1	27	19.10	0	7	4.19	5.25	67.80	34.12	7.34
226	1	29	20.45	1	5	6.50	3.56	67.80	31.59	0.82
227	1	36	20.40	0	8	5.75	6.94	66.20	22.36	8.76
228	1	35	23.88	0	6	7.18	4.82	65.50	23.06	1.89
229	1	31	31.25	1	11	7.94	13.79	65.20	26.15	5.11
230	1	35	21.48	1	5	7.12	3.29	65.10	5.71	3.03
231	1	24	23.51	1	8	6.00	9.00	63.00	57.00	3.53
232	1	29	28.19	0	15	9.05	9.13	60.00	49.67	2.93
233	1	26	24.00	0	3	8.00	3.00	59.00	31.00	1.00
234	1	30	21.36	0	12	6.61	4.71	57.30	33.69	4.31
235	1	31	20.55	0	6	8.16	5.03	56.70	15.28	2.65
236	1	26	21.63	0	10	5.12	6.61	54.20	46.92	11.61
237	1	34	23.31	1	4	9.94	4.07	53.90	20.80	0.11
238	1	32	23.92	1	7	7.22	5.32	52.00	23.10	2.10
239	1	39	21.61	1	5	7.66	10.29	50.20	62.33	4.83
240	1	35	18.83	1	6	9.00	5.00	50.00	23.00	1.65
241	1	43	20.57	1	2	8.86	4.48	49.60	34.45	0.02
242	1	30	21.23	1	10	7.00	5.00	49.00	38.00	3.53
243	1	37	28.08	0	7	5.00	4.00	48.00	18.00	0.87
244	1	33	23.80	1	5	6.49	3.99	47.30	3.56	2.04
245	1	27	15.94	0	10	6.00	6.00	47.00	28.00	2.06
246	1	35	23.73	1	6	7.00	3.00	47.00	32.00	1.50
247	1	36	20.96	0	3	11.05	4.94	47.00	36.67	1.25
248	1	39	27.73	1	4	11.38	2.30	46.40	13.63	0.77
249	1	37	28.23	0	7	4.91	4.59	45.50	34.01	0.73
250	1	26	20.70	0	8	8.63	1.95	45.10	27.35	1.32
251	1	25	23.07	0	4	6.82	4.10	44.80	34.39	3.00
252	1	25	18.96	0	5	6.20	3.86	44.40	19.63	2.53
253	1	27	24.22	0	6	5.64	3.52	44.20	19.29	2.88
254	1	35	24.03	0	6	7.00	2.00	44.00	11.00	3.53
255	1	35	21.48	0	6	7.00	2.00	44.00	55.00	2.59
256	1	37	27.82	1	4	7.77	2.15	44.00	30.76	1.19
257	1	24	24.44	1	5	8.62	7.89	43.00	22.63	1.70
258	1	33	28.44	1	8	6.95	4.29	42.60	21.80	3.00
259	1	28	24.17	0	9	9.08	2.07	41.90	43.20	0.56
260	1	34	20.81	1	4	5.47	4.92	41.20	21.56	6.37
261	1	35	18.23	0	4	9.00	9.00	41.00	27.00	3.53
262	1	30	23.67	1	7	5.90	3.14	40.30	11.69	2.30
263	1	34	27.64	0	6	3.02	3.71	40.30	11.69	3.07
264	1	30	26.37	0	8	6.68	3.54	40.00	15.43	1.40
265	1	31	20.28	1	6	8.00	5.00	40.00	12.00	3.53
266	1	40	24.39	1	11	6.00	5.88	39.30	23.73	14.33

267	1	35	21.23	0	5	7.07	3.01	39.10	10.39	2.98
268	1	36	23.00	0	8	8.00	8.00	39.00	45.00	3.95
269	1	25	17.97	0	10	7.36	5.44	38.30	28.30	5.13
270	1	29	29.76	0	10	5.29	5.71	38.00	24.79	9.21
271	1	27	22.68	0	11	8.13	2.73	37.40	20.65	4.94
272	1	29	19.53	0	4	6.16	2.57	37.30	28.20	1.31
273	1	29	19.53	1	8	5.39	4.38	37.10	24.42	16.75
274	1	30	21.48	1	7	4.80	5.37	36.90	19.31	5.32
275	1	37	23.73	1	8	8.44	4.78	36.90	18.75	1.48
276	1	36	20.55	1	6	6.40	3.53	36.60	20.68	5.08
277	1	32	29.30	1	6	6.01	2.67	36.10	34.00	5.28
278	1	35	36.05	1	4	10.78	2.08	36.10	14.46	0.82
279	1	36	29.24	0	4	5.17	2.99	35.80	28.41	0.02
280	1	27	24.61	0	6	5.45	3.09	35.20	23.30	1.10
281	1	23	23.44	0	9	7.44	3.93	35.10	21.99	7.25
282	1	26	23.51	0	6	5.87	2.32	35.10	27.94	2.27
283	1	38	22.64	1	4	6.45	3.12	34.20	17.84	1.28
284	1	40	29.97	1	9	5.00	4.00	34.00	24.00	3.61
285	1	24	25.39	1	8	5.91	3.08	33.80	10.22	7.39
286	1	32	20.32	0	3	6.32	3.61	33.40	21.05	2.69
287	1	25	21.48	1	6	6.00	4.00	33.00	30.00	1.19
288	1	41	24.44	0	6	7.08	4.37	32.10	18.55	0.37
289	1	26	17.36	1	6	7.00	5.00	32.00	34.00	3.53
290	1	32	22.06	1	6	10.00	3.00	32.00	25.00	1.31
291	1	27	20.25	0	5	7.03	7.98	31.30	25.50	8.91
292	1	26	21.30	0	11	6.32	4.81	31.00	43.19	8.68
293	1	27	24.09	1	12	6.00	4.00	31.00	31.00	3.98
294	1	31	21.48	0	6	7.00	4.00	31.00	21.00	3.36
295	1	33	23.93	1	5	8.44	4.28	31.00	29.17	0.82
296	1	29	23.53	0	13	3.97	4.95	30.70	50.61	4.98
297	1	34	21.36	1	6	8.75	4.49	30.70	21.36	6.19
298	1	22	22.77	1	18	5.00	6.00	30.00	18.00	11.64
299	1	29	21.93	1	7	6.00	7.00	30.00	23.00	0.20
300	1	30	18.73	1	8	9.00	9.00	30.00	24.00	2.51
301	1	29	25.22	0	9	5.64	1.51	29.90	14.22	4.12
302	1	25	26.04	1	10	5.12	5.16	29.10	41.94	3.92
303	1	31	28.76	0	8	7.00	4.00	29.00	15.00	1.24
304	1	34	20.32	0	6	6.37	4.59	28.00	11.92	0.33
305	1	34	24.03	0	5	6.00	5.00	28.00	6.00	0.67
306	1	35	25.24	1	10	8.00	3.00	28.00	39.00	0.63
307	1	23	21.30	1	6	7.00	5.00	27.00	59.00	2.41
308	1	32	25.63	0	8	5.17	4.73	26.90	27.25	7.17
309	1	38	19.53	1	5	7.49	4.24	26.20	16.08	4.70
310	1	34	22.27	1	11	7.00	8.00	26.00	36.00	1.01
311	1	35	19.53	1	5	7.18	5.35	26.00	23.28	2.68
312	1	41	21.23	0	6	6.00	4.00	26.00	6.00	2.00
313	1	27	22.35	1	10	7.00	5.40	25.60	25.95	5.11
314	1	30	18.05	0	6	5.48	3.76	25.20	18.64	3.48
315	1	24	25.97	1	12	7.00	4.00	25.00	27.00	3.87
316	1	40	24.17	0	14	10.00	4.00	25.00	17.00	0.17
317	1	28	20.82	0	12	7.78	6.81	24.70	35.68	6.25
318	1	28	21.19	0	6	8.22	4.50	24.40	16.26	0.47
319	1	30	23.33	1	12	6.00	7.00	24.00	39.00	3.53
320	1	38	22.21	1	9	12.00	6.00	24.00	40.00	0.73

321	1	32	25.96	1	3	7.50	3.04	23.80	17.65	0.56
322	1	29	19.92	0	3	9.66	3.76	23.60	17.02	3.01
323	1	38	25.78	1	6	6.67	4.12	22.80	26.52	1.54
324	1	24	23.53	1	10	5.00	4.00	22.00	28.00	2.58
325	1	37	25.96	1	4	7.10	4.51	22.00	15.70	4.91
326	1	23	20.42	0	9	6.51	5.89	21.80	29.02	10.00
327	1	34	27.64	1	5	5.69	2.87	21.20	30.29	6.87
328	1	23	19.78	1	3	7.00	4.00	21.00	18.00	0.29
329	1	27	20.28	1	8	5.00	4.00	21.00	10.00	3.79
330	1	30	26.49	1	4	10.00	3.00	20.00	26.00	0.31
331	1	39	27.06	1	8	5.04	4.91	17.50	31.09	3.41
332	1	33	26.11	1	4	7.00	4.00	16.00	22.00	1.69
333	1	37	24.22	0	6	5.00	4.00	16.00	16.00	2.37
334	1	26	25.00	1	6	7.00	4.00	15.00	22.00	0.57
335	1	24	27.18	0	9	5.10	3.15	11.90	18.97	2.73
336	1	33	25.04	0	8	6.00	3.00	11.00	14.00	2.38
337	1	30	20.93	0	5	19.32	3.99	10.00	16.53	0.74
338	1	33	22.48	1	6	8.00	5.00	9.00	12.00	3.53
339	1	29	25.97	0	10	5.00	5.00	8.00	14.00	3.96
